# Strepsirrhine Primate Training Programs in North American Institutions: Status and Implications for Future Welfare Assessment

**DOI:** 10.3390/ani11082462

**Published:** 2021-08-21

**Authors:** Gloria Fernández-Lázaro, Meg H. Dye, Christie Eddie, Gina M. Ferrie

**Affiliations:** 1 Animal Welfare Research Group and Friends of Thoreau Program, Franklin Institute, University of Alcalá, Alcalá de Henares, 28801 Madrid, Spain; 2 Didactic Department of Experimental, Social and Mathematical Science, Faculty of Education, Complutense University of Madrid, 28040 Madrid, Spain; 3 Duke Lemur Center, Duke University, Durham, NC 27708, USA; meg.dye@duke.edu; 4 Omaha’s Henry Doorly Zoo & Aquarium, Omaha, NE 68107, USA; christiee@omahazoo.com; 5 Disney’s Animals, Science and Environment, Lake Buena Vista, FL 32830, USA; Gina.M.Ferrie@disney.com

**Keywords:** animal welfare, primates, strepsirrhine, training, behavior management, operant conditioning

## Abstract

**Simple Summary:**

Training techniques are based on operant conditioning learning (the behavior is modified by its consequences). In many nonhuman primate species, they have been used to influence animals to perform specific behaviors voluntarily and cooperate with routine husbandry and veterinary procedures. However, the information regarding the suborder of strepsirrhine primates (lemurs, lorises, and galagos) is scarce. We assessed the development and current status of training programs with these species in North American institutions through an online survey. We collected information related to training program details; animals, behaviors, and techniques; the evaluation process; and the impact of training. Seventy-one organizations completed the survey, with results showing that 97% trained their strepsirrhines with the main objectives of husbandry and veterinary care (around 80%). Sixty-eight percent of organizations did not report any risk in training these species. The benefits reported include increases in positive human–animal interactions, psychological well-being, and staff awareness of animal behaviors, supporting the success of these programs in providing optimal care for these nonhuman primates. However, we need to improve our understanding of the impact of training on the welfare of strepsirrhine primates, and we hope that the data offered in this survey can help in this future assessment.

**Abstract:**

Many articles have shown the benefits of operant conditioning training techniques in the care and welfare of several species of nonhuman primates; however, the information regarding their use in strepsirrhine species is scarce. We assessed the development and current status of training programs with these species in North American institutions. An online survey was distributed through members of the Association of Zoos and Aquariums using a multiple-choice format. We collected information related to training program details; animals, behaviors, and techniques; the evaluation process; and the impact of training. Seventy-one organizations completed the survey, with the results showing that 97% of respondents trained their strepsirrhines with the main objective of husbandry and veterinary care (around 80%). Sixty-eight percent of organizations did not report any risk in training these species. The benefits reported include increases in positive human–animal interactions (97%), psychological well-being (88%), and staff awareness of animal behaviors (90%)**.** However, a multi-dimensional approach to measure the efficacy of training could provide a deeper understanding of its impact on the welfare of strepsirrhine primates. We hope that the data offered in this survey can help in this future assessment.

## 1. Introduction

The use of operant conditioning has been proven to be very useful in the management and welfare of animals in managed care [1,2,3,4]. The benefits of training have been grouped in four main areas, with many examples of each described in studies of nonhuman primates: (1) improved husbandry and medical care through voluntary animal cooperation in procedures [5,6,7,8]; (2) enhanced social management [9,10,11]; (3) improved psychological well-being [12,13,14], and (4) increased options for environmental enrichment [15]. Numerous studies have successfully applied operant conditioning in several species of nonhuman primates, including chimpanzees (*Pan troglodytes*) and rhesus macaques (*Macaca mulatta*) [16,17], bonobos (*Pan paniscus*) and Sumatran orangutans (*Pongo abelii*) [18], marmosets (*Callithrix* spp.), tamarins (*Saguinus* spp., *Leontopithecus* spp., *Callimico* spp.), and saki monkeys (*Pithecia pithecia*) [19,20], mangabeys (*Cercocebus atys atys*) [8], western lowland gorillas (*Gorilla gorilla gorilla*) [21,22], owl monkeys (*Aotus* spp.) and squirrel monkeys (*Saimiri* spp.) [23], mandrills (*Mandrillus sphinx*), gibbons (*Nomascus leucogenys*), siamangs (*Symphalangus syndactylus*) and colobus monkeys (*Colobus guereza*) [24], and baboons (*Papio* spp.) [25,26]. However, the information regarding the application of training techniques in strepsirrhine species (lemurs, lorises, and galagos) is scarce. Thus, we lack information about species-specific training, how training is being used, possible benefits or problems, and how these programs are monitored and evaluated for an entire suborder of primates.

Several studies have described strepsirrhine species being successfully trained to participate in projects investigating sensory perception and cognition [27,28,29] and locomotion [30,31,32]; however, there is very little information in the scientific literature about the status of training programs for these species or the effects on the welfare of this suborder of nonhuman primates. Communications in animal behavior and animal keeper conferences have shown that these species are being trained in zoos and research facilities to address husbandry and veterinary concerns [33,34,35,36,37,38,39,40], and three recent publications have given more insight into these topics: (1) a handbook of primate behavioral management, including an entire chapter dedicated to strepsirrhines, which gives information on how training is being used in these species to help in husbandry, veterinary medicine, and research [41]; (2) another book dedicated to zoo animal learning and training, giving examples of how positive reinforcement training (PRT) is being applied to enhance the welfare of nonhuman primates in zoological collections, including some strepsirrhine species [42]; (3) a study highlighting the lack of information on training in these species, which explored the role of an isolation PRT program on the well-being of ring-tailed lemurs (*Lemur catta*) [43]. The results of these publications support that training may play a crucial role in the management of these nonhuman primates in human care. Spiezio et al. [43] also highlighted the importance of research in these areas, helping to improve the husbandry standards for animals in managed care by designing adequate species-specific training programs (something previously noted, for example, by Schapiro et al. [16]).

In fact, training may not only differ by species but among individuals. Factors such as age, sex, social rank, early experience, housing conditions, and personality (defined as individual differences in behavior that are consistent over time and situations [44]) can affect training success and the welfare impact [45,46,47,48,49]. For example, for chimpanzees, males are more likely to participate in initial training sessions to voluntarily submit to a blood glucose test than females [17] but adult female chimpanzees require considerably fewer ongoing training sessions than adult males to move voluntarily into an indoor enclosure [48]. Additionally, chimpanzees that rate higher on the personality factor “openness” are more likely to participate in training sessions involving blood glucose testing [17], and, for long-tailed macaques (*Macaca fascicularis*), the personality trait “activity” is associated with training success [47]. In fact, in that study, training success was better explained by personality than by social rank. Other articles have reported that allowing titi monkeys to observe cage-mate training sessions may enable them to be trained more rapidly via increased familiarity with the training task through additional exposure [19]. Concretely, authors in that study reported that the introduction of less timid species from the training program (*Saguinus imperator* and *Callithrix kuhlii*) allowed Bolivian grey titi monkeys (*Callicebus donacophilus*) to learn by observation [19]. Therefore, further research on these factors will help to improve training programs and provide optimal care for nonhuman primates in managed care [50].

The aim of this study is to assess the development and current status of training programs with strepsirrhine species housed at facilities in North America (accredited by the Association of Zoos and Aquariums (AZA)), assess if the staff members responsible for training believe training elicits similar benefits in other nonhuman primates, and look to the future of training with strepsirrhine primates in the context of animal welfare assessments. Additionally, we seek to collect information on the current status of assessing the impact of training on animal welfare.

## 2. Materials and Methods

We focused this study on the North American region and specifically with institutional members of AZA for several reasons. First, AZA includes two institutions dedicated entirely to this suborder of nonhuman primates: the Duke Lemur Center, the world’s largest colony of strepsirrhine primates outside of Madagascar, who have had a successful training program implemented for more than 10 years [51,52], and the Lemur Conservation Foundation. Second, AZA is the second-largest regional association in the world (after the European Association of Zoos and Aquariums) holding strepsirrhine species, according to the Zoological Information Management System (ZIMS; as of December 2020). Finally, given the space assessment conducted by the Prosimian Taxon Advisory Group (PTAG) from the AZA in 2018, 125 AZA organizations hold strepsirrhine species. Collectively, these institutions hold a total of 26 species of strepsirrhines, including one species (*Loris tardigradus*) which is not maintained in Europe [53].

To gather information for this study, we developed an online survey. We modeled our survey after the similar methodology of published research, including the welfare evaluation of the Nile hippopotamus (*Hippopotamus amphibius*) in North American zoos and aquariums [54] and the evaluation of behavioral management and environmental programs for laboratory primates [55,56,57]. Our survey, consisting of 23 questions (note that this is an approximation because some institutions responded to only 2 questions if they did not train their strepsirrhines or to 24 or 25 questions due to the structure of the survey), was created using Google forms. It used a multiple-choice format and was divided into three sections, consisting of questions related to (1) training program details, (2) animals, behaviors, and techniques, (3) the evaluation process, and the impact of training (Supplementary S1).

We included some questions based on previous surveys on training nonhuman primates in laboratories [57] and added additional questions relevant to the taxa. After being reviewed and approved by the PTAG Steering Committee, the survey link was distributed by email through the AZA-accredited and -certified facilities holding these nonhuman primate species. We requested that the staff member responsible for training strepsirrhine species complete the online survey. In order to be aware of the required information prior to completing the survey, we included a pdf with the questions that would be asked on the survey (Supplementary S1). Participants were informed that only one survey per institution would be needed and that the information collected may be used for publication in a research journal and/or academic conferences. The name of the institutional affiliations would not be included in analyses and would remain anonymous.

The response collection period lasted three months, from mid-October 2019 to mid-January 2020. After that, the responses were evaluated and checked individually for repetition or identification of data in need of clarification. Descriptive analyses were done, providing frequencies and percentages of response for the questions. Since many questions allowed the selection of multiple responses, many totals sum up to more than 100%. This research adhered to the American Society of Primatologists (ASP) Principles for the Ethical Treatment of Nonhuman Primates.

## 3. Results

### 3.1. Responses and Institutions

From the 81 responses collected, one was received 99% incomplete and, therefore, excluded. Seven institutions had two respondents and one had three. In order to clarify the answers that did not match, these institutions were contacted a second time to clarify their duplicate responses. Overall, 71 unique organizations completed the survey (62 with a rate of completion of 100% and 9 institutions with 96%). According to the 2018 AZA PTAG space assessment, the survey was answered by 57% of the AZA institutions holding strepsirrhine species.

### 3.2. Training Program Details

Two institutions did not train strepsirrhine primates due to, respectively, lack of time and training time given to higher priority species. The other 69 institutions trained strepsirrhine primates: 85.5% as part of a formalized training program and 14.5% by keepers interested in training but without a formalized program at their facility.

Nearly half of the institutions (46%) have been actively training strepsirrhine primates between five and 10 years, 29% have been actively training between 10 to 20 years, and 25% have been training for less than five years.

The most frequent objectives of the training program were husbandry (87%) and veterinary care (78%), while the least frequent were research (3%) and education (16%). The majority of institutions (80%) had only full-time employees training strepsirrhine primates, while 20% reported a combination of full-time and part-time employees. In 6% of facilities, volunteers and students assisted with the training sessions. 

Two resources were identified as the most helpful (>50% of responses) either in establishing or advancing the training program (Table 1): in-house experience and staff meetings and discussions. More than 70% of institutions found them to be the most valuable resources for the formalization of the program. In advancing training programs, these two resources were most helpful for increasing the number of trainers (>70% of the responses) while still being a valuable resource for expanding training to more individual animals (60–70% of the responses) or more species (46–55% of the responses). Other useful resources listed by participants were access to animal training articles, animal training books, and social media from animal training forums. 

### 3.3. Animals, Behaviors, and Techniques

The majority of institutions (78%) trained a maximum of 10 individual strepsirrhines. In fact, 41% trained less than 5 individuals, and only 1 institution trained more than 50 individuals. 

With regard to the training staff, the majority of institutions (96%) reported having less than 10 people training strepsirrhine species, while 60% reported less than 5 people. On average, trainers spent less than 30 min training strepsirrhines per day in 85% of the institutions. Individual animal training sessions lasted between 5–10 min in 54% of institutions and 5 minutes or less in 35% of institutions. Individual animals had between two to five training sessions per week in 60% of the institutions, less than two sessions a week in 25%, and more than five sessions a week in 15% of institutions.

A total of 23 strepsirrhine species are being trained in AZA institutions (Table 2). The species most commonly housed and trained is the ring-tailed lemur (*Lemur catta*) in 88% of the institutions, followed by the ruffed lemurs (*Varecia rubra* in 40% and *Varecia variegata* in 35%). Within the nocturnal species, the most commonly trained is the Pygmy slow loris (*Nycticebus pygmaeus*) in 16% of the institutions, followed by the aye-aye (*Daubentonia madagascariensis*) in 7% of respondents. 

Eleven institutions did not report any challenges in training strepsirrhine species, while 58 cited a variety of challenges with specific species. The most commonly named (n = 20) was difficult environmental conditions (including enclosure space or low-light conditions), with 50% being associated with nocturnal species (families Cheirogaleidae, Daubentonidae, Galagidae, and Lorisidae) and 25% with ring-tailed lemurs. Additionally, being shy or skittish (n = 16), slow training progress (n = 15), or being unmotivated (n = 11) were identified as challenges for certain species (Table 3). Additional challenges indicated in participant notes included separating the animals, training in mixed-species exhibits, disruption of the social structure, and training during the breeding season (n = 18). Additionally, 14 institutions highlighted that there was no training challenge concerning the species but rather with the personality (n = 7) or age (n = 4) of the individuals. Participants also reported health-related management concerns with individuals of some species (n = 3), which made them difficult to train. Medical challenges included the restraint of diabetic ring-tailed lemurs for injections and Coquerel’s sifaka (*Propithecus coquereli*) for contraception injections during the breeding season.

Regarding training techniques (Appendix A), all of the institutions used positive reinforcement to encourage the desired behavior. The utilization of jackpots was identified by 64% of participants and vocal encouragement by 39%. When looking to discourage a behavior, nearly half of the institutions (55%) used one method while the other half (45%) used two methods. The utilization of least reinforcing stimulus (LRS) was used most commonly (75%), while the use of time-outs was used by 54%. Collectively, 16% identified other methods to discourage behavior, including not offering the reward, redirecting the behavior, and asking for an incompatible behavior. To shape a behavior, all of the participating institutions used successive approximations to achieve their behavioral goal. While using approximations, 93% of the facilities use baiting and 68% active desensitization/counter-conditioning. Free shaping or scanning was used in 39% to elicit the desired behavior, while 17% used modeling to shape the behavior. The most common behaviors trained with strepsirrhine species are shown in Table 4, with the most frequent (94%) categorized as “the basics” for husbandry (bridge, station, target, follow target, point follow), scale training (93%), kennel training (88%), and shifting/separation (78%).

### 3.4. Evaluation Process and Impact of Training

Most institutions did not report any risks in training strepsirrhine species (68%), although 16% of them cited “increased staff injury” and “changes in animals’ social structure”. Additional risks to conducting training sessions included “human habituation” (6%), “changes in animal behavior” (7%), “increased animal injury” (6%), or “decreased animal care due to time spent training” (7%). No institutions indicated “monetary expenses” or “interference with research, education, or management” as a concern.

Almost all of the responding institutions (97%) identified the increase in positive human–animal interactions as a benefit of having a strepsirrhine training program. Other benefits that received a high response (>87%) included “increased animal psychological well-being”, “increased efficiency in husbandry management”, “increased efficiency in veterinary care”, and “increased staff awareness of animals’ behaviors” (Table 5). 

Regarding training session record keeping, there was not a unique method that institutions clearly used (Table 6). Nearly half of them (47%) marked electronic sheets, and 41% used paper sheets to record animal training sessions. Additionally, 46% of the respondents used a combination of two or more methods. To ensure consistency and transfer of trained behaviors, 78% of the institutions reported having regular meetings to share information; 71% recorded data and documented information such as the number and duration of sessions, shaping-plan steps trained, name of the trainer, and animal´s response. Less than half of the institutions (42%) had a criterion to determine if the animal had learned the shaping plan step, and only 19% reviewed data from the sessions and created documents to ensure the efficiency of the program; even less (12%) invite speakers or staff to attend professional meetings.

When asked if their institution evaluates the impact of training on the well-being of these nonhuman primates, 70% of institutions indicated they did not. From the ones that did (n = 21), the most utilized methods were behavioral measures (95%), followed by video recordings (10%). Only one institution used physiological measures.

**Figure 1:**
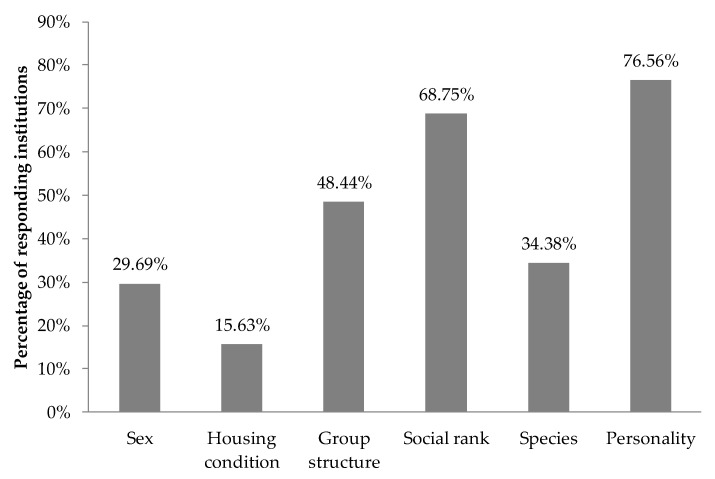
Variables reported by North American institutions (n = 64) that lead to differences in training success in strepsirrhine primates.

Finally, we also asked if the staff noticed differences in training success according to any variable (Figure 1), and although personality was the most cited (77% of responses), the majority of institutions (94%) did not have a formal process to assess the personality or temperament of the strepsirrhines trained or those who were going to be trained. From the four institutions that did, all used cumulative observations (ratings are based on the knowledge and experience that each rater has accumulated because he or she has known the animal). Nevertheless, two of these organizations also reported using naturalistic observations (coding or rating the animals over a specific period of time based on their ordinary daily behavior), and the other two used behavioral coding; one also used behavioral tests (coding or rating the behavior of the animal in response to a particular situation or experiment, e.g., novelty, aversive stimuli, mirror test). No institutions responded that they use rating personality traits (people familiar with the animals rate them on a set of predefined traits or adjectives on a scale, for example, from strongly represented to not represented).

## 4. Discussion

To our knowledge, this is the first study to collect information on strepsirrhine primate training programs in North American institutions. The high level of survey completion rate of the participant institutions (between 96–100%) could represent the interest in the topic as other surveys with similar formats received lower completion rates [54,55,56]. In fact, around 25 multiple choice questions could have potentially been too many for busy zoo staff members to answer completely [54]. However, the high survey completion rate reflects the great effort and interest from the participating institutions. It is possible that explaining how the online survey works, distributing the questions in a pdf format ahead of time, grouping the questions clearly in three sections, and distribution through the AZA PTAG decreased factors that are commonly cited to affect response rates, including having a sponsoring organization, question order, question display, contact delivery modes, and pre-notification [58,59]. Although we covered only 57% of the AZA institutions that hold strepsirrhine species, the average response rates of online surveys in 2008 were calculated to be around 11% to 15% [60], and it has been suggested to have dropped even further since then [58]. Again, this could represent the attractiveness of the topic, and, in the future, it would be illuminating to expand the survey to other regions that hold these species. In particular, it would be interesting to survey facilities in Europe due to the high number of zoos and aquariums that manage these species, including eight species not housed in North America [53]. 

### 4.1. Training Program Details, Animals, and Techniques

The high percentage of participant institutions that train strepsirrhine primates (97%) for husbandry and veterinary care (around 80%) suggests that these techniques are mainly being applied to enhance welfare through the cooperation of animals in many procedures, as cited previously in many other nonhuman primate species [4,5,11]. In fact, with 92% of facilities doing scale training, 88% kennel training, and 78% separation training, with an average time in most of them (85%) of less than 30 min training strepsirrhines per day by individual staff members and 89% reporting training sessions under 10 min, the results based on staff responses reveal that behavioral goals can be achieved with relatively short time investment. Moreover, advanced behaviors, such as syringe training (45%) and injection training (38%), are emerging in multiple training programs, revealing that, although strepsirrhine training has not advanced to the same complexity as with larger nonhuman primates, the interest and skills to do so are making great progress. With 7% of facilities training for blood draw and 14% training for voluntary restraint, our survey reveals the development and trajectory for expanded opportunities that training strepsirrhines for voluntary participation is having in the behavioral management of these species. 

Other benefits reported from these programs include increases in positive human–animal interactions, psychological well-being, and staff awareness of animal behaviors, as previously stated for other species [2,12,13]. All these benefits, together with most organizations not reporting any risk in training strepsirrhine species (68%), confirm the success of these programs (as perceived by the staff) in contributing to the optimal care given to these nonhuman primates. It is also remarkable that no institution found monetary expenses as a concern, which is in contrast to what was reported for training programs for nonhuman primates in laboratories [57]. Moreover, finding that 75% of institutions have been actively training strepsirrhine primates for more than five years (with 29% of them training them for more than 10 years) highlights the expansion that these techniques are having in zoos and research centers. These results also confirm a change of tendency from what was reported for North American zoos and related facilities in 2010, where the vast majority of institutions rarely or never conducted training with lorisid primates [61]. In our survey, 15 institutions trained lorisid primates with Pygmy slow loris (*Nycticebus pygmaeus*), the most commonly trained. Given that 20 facilities in North America house pygmy slow loris [62], our survey indicates that a minimum of 55% of institutions with pygmy slow lorises in North America participate in a training program. Similarly, 86% of institutions that house aye-ayes (*Daubentonia madagascariensis*) in North America [63] participate in a training program, with six of seven institutions indicating the species as one participating in their training program. 

It was not surprising to find that ring-tailed lemurs (*Lemur catta*) were the species most commonly trained. Currently, 101 AZA facilities house ring-tailed lemurs [64], making it the most numerous species of any strepsirrhine primate in North America. This diurnal species, with others such as ruffed lemurs (genus *Varecia*) and some *Eulemur* species, are highly social and live in multi-male/multi-female social groups of about 6–30 individuals [65]. These species are usually maintained in larger groups and/or with mixed species, and behavioral management can be challenging, especially during breeding seasons, as reflected in our survey results. However, PRT has been documented as a useful tool to assist with behavioral management during challenging situations. Voluntary separations have been documented to eliminate behavioral indicators of fear or anxiety and teach individuals to remain calm for the duration of the separation [43,51]. 

### 4.2. Evaluation and Impact of Training

All the institutions, except three, document their training in some way, and the majority of them (70–78%) ensure consistency through meetings or sharing recorded data. With a growing understanding of the role of training in promoting opportunities for choice and positive engagement, documentation is important for program assessment and evidence-based decisions, as previously stated [57,66]. However, the lack of a unique method to record it or a unique platform, as evidenced by our survey, can make the evaluation of training programs difficult. In fact, only a small percentage of participants (19%) reviewed data from training sessions. There may be several reasons for this, with the main factor, as assumed by the authors, being the lack of time. Transcribing data from paper records to a computer-based analysis platform (e.g., Excel, ZIMS Care and Welfare, statistical or behavior analysis program) can be extremely time-consuming depending on the size of the training program, both in the number of trainers and/or individuals being trained. Records maintained in a digital database such as Excel will make analysis easier if in-house expertise is present to manipulate data and create graphs. While data analysis from training sessions can be challenging, progress is being made for user-friendly platforms that automatically create descriptive stats. Currently, a variety of tools, such as training templates in ZIMS, TRACKS, and other privately or individually developed software templates, are being developed and tested for the ease of data entry and output relevancy.

Another interesting result found in our survey was that although all the institutions used successive approximations to achieve behavioral goals, only 42% of them had criteria to determine if the animal has learned the shaping plan step. Again, we think that this can be related to the difficulties in analyzing training data and the lack of a unique system to record training sessions. However, adding the completion date of individual approximations as a trainer works toward the final goal combines two important elements of a training program, shaping plans and training records while also creating an additional output measurement to assess the total amount of time for an individual to learn a behavior and the duration of time to learn each step of the shaping process. Therefore, we recommend including it in the documentation because it can lead to the improvement of the analysis of training sessions, creating, along the way, opportunities to discuss training techniques and shaping plans. When discussing a challenging training situation, managers and trainers can look at the training data to identify when an animal’s learning has plateaued or regressed and adjust accordingly. Data analysis may also play an important role when assessing inter-species training goals and species-specific traits that contribute to or inhibit the successful completion of husbandry behaviors.

When asked about training differences, respondents found the personality of the individuals as one of the main factors (77%) that can influence training success, something that has also been previously suggested for many other nonhuman primate species [17,45,46,47,67]. In fact, institutions in our survey also identified several personality traits as challenges when training strepsirrhine species, such as being “shy or skittish”, “hyperactive”, “hyper-reactive”, or “easily distracted”. However, the majority of them (94%) did not have a formal process to assess the personality of the individuals. Incorporating a formal process to evaluate personality, combining behavioral coding and trait rating methods [44,68], may not only provide a more thorough insight into these differences to adjust shaping plans, but it can also be applied in many areas to improve the welfare of nonhuman primates [45,68,69,70,71]. Following these applications, several zoological associations, for example, the British and Irish Association of Zoos and Aquariums (BIAZA), have developed guidelines that include explanations of how to measure and analyze the personality and benefits that this research brings to different areas of health, management, and conservation of zoo animals [72]. Additionally, there are books dedicated entirely to personality or temperament in nonhuman primates [73], and, specifically for strepsirrhine species, several studies have measured it with behavioral coding identifying traits on a spectrum of boldness to shyness ([74] with *Otolemur garnetti*; [75,76] with *Microcebus murinus*), while others have combined behavioral coding and trait rating ([77] with *Nycticebus pygmaeus*).

Another factor found to influence training, supported by our survey results, was the species. For example, tamarins were reported to more rapidly approach trainers and learn behaviors than marmosets [19], and squirrel monkeys (*Saimiri* spp.) learned a simple “target” behavior (touching a stationary object when presented) significantly faster than owl monkeys (*Aotus* spp.), although these species did not differ in the amount of time required to train subsequent behaviors [23]. For strepsirrhines, a study developed at the Duke Lemur Center with the *Eulemur* species by the first author (G.F.L.), supervised by the second author (M.H.D.), also found significant differences related to the species [78]. This research on 14 lemurs was performed as part of a Ph.D. dissertation, which found that red-collared lemurs (*Eulemur collaris*) approached trainers and progressed in training (following a target around a short, simple obstacle course to complete an S shape) more rapidly than the other study species (Supplementary S2). Additionally, white-fronted brown lemurs (*Eulemur albifrons*) took the longest to approach trainers and showed the slowest progress in training, but no sex differences or partner effects were found (Supplementary S2). Other researchers have also reported training differences between lemur species [79]. Ring-tailed lemurs (*Lemur catta*) ceased responding earlier than brown lemurs (*Eulemur fulvus*) during the first extinction phase (i.e., the response is lost over time when a reward is no longer provided) and attained higher response rates during subsequent reinforcement sessions. However, with only one subject representing each species, it would be premature to conclude that a species difference has been demonstrated in that study. Additional data on training variations in lemur species may allow us to see if these results remain and to test whether differences persist in longer studies that include the training of subsequent behaviors. Longer studies would also allow us to test whether or not there is species variation in persistence to learn a new task, a trait that has been shown in red-fronted lemurs (*Eulemur rufriforns*) to be important for individual success during innovation [80], which could play an important role in training success.

Our survey results also revealed that although the application of training to promote positive animal welfare through voluntary participation is in line with best practices, as outlined in AZA guidelines and other nonhuman primate training programs [4,5,11], most of the organizations (70%) did not monitor the impact of training on the well-being of these species. It is true that a positive indicator of staff perception of training programs with strepsirrhine species is that these programs contribute to the optimal care of the species; however, as Melfi and Ward stated [81], the welfare impact of training has to be based on the empirical monitoring of its application. As an extension of the challenge of analyzing training records, the creation of assessment tools is an area of rapid expansion in the zoological community, as we have stated previously with personality. As animal welfare refers to the physical health, behavior, and emotional state of animals, measuring it requires a multi-dimensional approach combining different indicators [82]. This can be complex, but we already have examples in strepsirrhine primate species that combine behavioral observations, personality ratings, and physiological measures [77,83]. With advanced husbandry behaviors increasing with strepsirrhine species, evidence-based physiological measures may become more prevalent. Voluntarily collected biological samples, such as blood, urine, and saliva, would help validate species-specific behavioral indicators currently used to describe good welfare and a positive affective state. The validation of the positive effects of choice, control, and engagement for an individual participating in a training program would benefit from a combination of both behavioral and physiological indicators. Utilizing a multi-dimensional approach to measure the efficacy of training to reduce negative stressors and increase positive effects could provide a deeper understanding of the impact of training on the welfare of strepsirrhine primates.

### 4.3. Recommendations

The authors strongly recommend that facilities that currently do not keep training records create a system to do so as the analysis of behavior is critical for problem-solving and monitoring an individual animal’s progress. For facilities that currently do have written documentation of their training sessions, additional fields of data collection and other actions may be helpful. For example, adding the completion date of individual approximations, digitalizing records, and homogenizing training templates across institutions and/or platforms would allow the analysis of training data to be easier and faster.

Lastly, the incorporation of a formal process to evaluate the personality of individuals is recommended. Personality assessments can bring benefits not only to the training program when deciding on training techniques that suit an individual but also in animal welfare assessment, something that has to be improved by applying a multi-dimensional approach based on empirical monitoring.

## 5. Conclusions

Information on training nonhuman primates does not generally include strepsirrhine species. However, our survey results have revealed not only an increase in the number of strepsirrhine training programs in North America but an increase in the advanced behaviors that are being conditioned within the programs. While strepsirrhine training is catching up with other nonhuman training programs, the benefits reported by the respondents are similar to other species and confirm the success of these programs in contributing to the optimal care of these species. The creation of animal welfare assessments and specifically using training to promote good welfare is currently a discussed topic for many animals in human care. We hope that the data offered in this survey will help to guide the assessment of strepsirrhine training programs, refining the collection and utilization of training data to guide behavioral management and animal welfare decisions.

## Figures and Tables

**Table 1:** Most helpful resources detected in establishing or advancing the training program with strepsirrhine species.

Resources ^a^	Formalization of Program		Expansion to More Trainers		Expansion to More Species		Expansion to More Individual Animals	
	n_i_	%	n_i_	%	n_i_	%	n_i_	%
In-house experience	59	88.06	56	83.58	37	55.22	45	67.16
Outside consultant	25	37.31	14	20.90	8	11.94	7	10.45
Animal training workshops or conferences	30	44.78	18	26.87	16	23.88	15	22.39
AZA training community discussion board	13	19.40	8	11.94	9	13.43	4	5.97
Staff meetings and discussions	48	71.64	49	73.13	31	46.27	40	59.70
Training videos	19	28.36	17	25.37	10	14.93	12	17.91
Invited speakers	11	16.42	5	7.46	6	8.96	3	4.48

^a^ Total institutional responses = 67. Two institutions did not respond to this question. “n_i_” = number of institutions responding.

**Table 2:** Strepsirrhine species trained in North American institutions (n = 68).

Family	Species	Number of Institutions	% of Respondents
Cheirogaleidae	*Cheirogaleus medius*	1	1.47
	*Microcebus murinus*	2	2.94
Daubentonidae	*Daubentonia madagascariensis*	5	7.35
Galagidae	*Galago moholi*	6	8.82
	*Otolemur crassicaudatus*	0	0
	*Otolemur garnetti*	7	10.29
Indridae	*Propithecus coquereli*	9	13.23
Lemuridae	*Eulemur albifrons*	2	2.94
	*Eulemur collaris*	10	14.71
	*Eulemur coronatus*	6	8.82
	*Eulemur fulvus*	5	7.35
	*Eulemur macaco flavifrons*	8	11.76
	*Eulemur macaco macaco*	2	2.94
	*Eulemur mongoz*	9	13.23
	*Eulemur rubriventer*	1	1.47
	*Eulemur rufus*	1	1.47
	*Eulemur sanfordi*	1	1.47
	*Hapalemur griseus*	1	1.47
	*Lemur catta*	60	88.23
	*Varecia rubra*	27	39.71
	*Varecia variegata*	24	35.29
Lorisidae	*Loris tardigradus*	0	0
	*Nycticebus bengalensis*	1	1.47
	*Nycticebus coucang*	0	0
	*Nycticebus pygmaeus*	11	16.18
	*Periodictius potto*	3	4.41

**Table 3:** Number of institutions reporting challenges in training Strepsirrhine primates.

Family	Species	Safety Concerns	Slow Progress	Shy or Skittish	Difficult Environmental Conditions	Hyperreactive	Hyperactive	Unmotivated	Easily Distracted/Hard to Focus
Cheirogaleidae	*Microcebus murinus*			1	1				
Daubentonidae	*Daubentonia madagascariensis*				1				
Galagidae			2	5	5				
Indridae	*Propithecus coquereli*				1			1	
Lemuridae	*Eulemur albifrons*							1	
	*Eulemur collaris*			1				2	
	*Eulemur coronatus*		1	1					
	*Eulemur fulvus*	1	1	1					
	*Eulemur macaco flavifrons*	1				1	1		1
	*Eulemur mongoz*		1					1	
	*Lemur catta*	1	7	5	5		1	3	1
	*Varecia rubra*		1		2	1	2	2	1
	*Varecia variegata*	1				2	2		
Lorisidae	*Nycticebus pygmaeus*		1	2	2			1	
	*Periodictius potto*		1		1				
No species indicated					2				
	Total	4	15	16	20	4	6	11	3

**Table 4:** Behaviors trained with strepsirrhine species in North American institutions (n = 69).

Behaviors Trained	Number of Institutions	% of Respondents
Basics: bridge, station, target, follow target, point follow	65	94.20
Shape recognition	10	14.49
Scale training	64	92.75
Kennel training	61	88.41
Shifting/separation (following target or hand cue)	54	78.26
Hang or other posture training for physical examination	40	57.97
Voluntary restraint to be transported short distances	10	14.49
Syringe training to administer medication or fluids	31	44.93
Palpation or manipulation for vet examinations	38	55.07
Injection training	26	37.68
Positional and duration control for ultrasound or X-rays	21	30.43
Blood sample	5	7.25
Infant removal	5	7.25

**Table 5:** Benefits reported of training strepsirrhine species in North American institutions (n = 69).

Training Benefits	Number of Institutions	% of Respondents
Increased animal psychological well-being	61	88.41
Increased positive human–animal interactions	67	97.10
Increased efficiency in husbandry management	60	86.96
Increased efficiency in veterinary care	62	89.86
Increased staff awareness of animals’ behaviors	62	89.86
Enhanced education of the public	36	52.17
Increased staff communication	31	44.93
Decreased stress-related behaviors	32	46.38
Decreased human-directed aggression	19	27.54

**Table 6:** Training documentation and record-keeping used in North American institutions (n = 68).

Training Documentation	Number of Institutions	% of Respondents
Paper sheets filled by trainers	28	41.18
Electronic sheets filled by trainers	32	47.06
Videos	21	30.88
Photos	15	22.06
There is no record keeping	3	4.41
Computer applications for training documentation (ZIMS, TRACKS)	18	26.47

ZIMS = Zoological Information Management Software (Minneapolis, MN, USA https://www.species360.org/products-services/zoo-aquarium-animal-management-software-2/ accessed on 20 August 2021). TRACKS^®^ sofftware (Colorado, AZ, USA https://trackssoftware.com/ accessed on 20 August 2021).

## Supplementary Materials

The following are available online at https://www.mdpi.com/article/10.3390/ani11082462/s1, Supplementary S1: Strepsirrhine primate training survey, Supplementary S2: Differences in training success in five *Eulemur* species.

## Appendix A

For a detailed explanation of training terms and techniques, please consult the following link developed by AZA and the American Association of Zoo Keepers (AAZK): https://static1.squarespace.com/static/57c607322e69cf3a289944bb/t/5f1703a1710f1d7d07d1b115/1595343777563/AZA+AAZK+Training+Terms+2019.pdf (accessed on 20 August 2021).
