# Supplementary material for: Strepsirrhine Primate Training Programs in North American Institutions: Status and Implications for Future Welfare Assessment
Source: Animals (Basel). 2021 Aug 21;11(8):2462. doi: 10.3390/ani11082462 (PMC8388707; doi:10.3390/ani11082462)
Supplement: Supplementary file 1 [file animals-11-02462-s001.zip › animals-1300893-supplementary/S1.pdf]

Supplementary File 1 (S1): Strepsirrhine primate training survey

The order of Primates is divided in two suborders: Strepsirhines and Haplorhines. Living strepsirhine primates include lemurs (Madagascar), lorises from Asia and pottos, galagos and angwantibos in Africa.

Questions are divided in three sections:
1) Training program details
2) Animals, behaviors and techniques
3) Evaluation process and impact of training

For all the questions there are multiple choice answers. Please mark all that apply.

1. Training program details

1.1. Do you train your strepsirrhine primates? This could be one individual or all in your collection.
   a) Yes, we have a formalized program
   b) Yes, there is keeper interest. We do not have a formalized program
   c) No, we do not train our strepsirrhine primates

If you answered “c” to 1.1, what is preventing you from implementing a training program?
a) Staff do not have time to train
b) Training time is given to higher priority species at our facility
c) No one on staff has experience initiating a training program
d) We do not have funding to bring in a training consultant
e) Other reason (please specify)

If you answered “a” or “b” to 1.1, what is the main objective of your training program?
a) Husbandry
b) Veterinary care
c) Research
d) Enrichment
e) Education
f) Animal welfare
g) Other reason (please specify)

1.2. What is the current employment status of individuals training the animals?
Please enter the percentage of trainers that fall into each category below

a) full time employees ______
b) part-time employees ______
c) volunteers ______

1.3. How long has been your facility actively training strepsirrhine species?

a) Since this year
b) Less than 2 years
c) Less than 5 years
d) 5 to 10 years
e) 10-20 years

1.4. What resources were most helpful in establishing or advancing your training program for strepsirrhine species? Please check the appropriate box(es) under each of the following categories:

i Formalization of program
ii Expansion to more trainers
iii Expansion to more species
iv Expansion to more individual animals

**Resource**	**i**	**ii**	**iii**	**iv**
a) In house experience				
b) Outside consultant				
c) Animal Training workshops or conferences				
d) AZA training community discussion board				
e) Staff meetings and discussions				
f) Training videos				
g) Invited speakers				
h) Other (please specify)				

2. Animals, behaviors and techniques

2.1. Approximately how many strepsirrhines are currently being trained at your facility?

a) <5
b) 6-10

c) 11-20
d) 21 - 50
e) >50

2.2. From the list below, please indicate which species you train. Choose all that apply:

Family	Species (common name)	
**Cheirogaleidae** (dwarf and mouse lemurs)	*Cheirogaleus medius* (fat tailed dwarf lemur)	
	Microcebus murinus (mouse lemur)	
**Daubnetonidae** (aye-aye)	*Daubentonia madagascariensis* (aye-aye)	
**Galagidae** (galagos)	*Galago moholi* (Moholi bushbaby)	
	Galago senegalensis (Senegal bushbaby)	
	Otolemur crassicaudatus (Brown greater galago)	
	Otolemur garnetti (Northern greater galago)	
**Indridae** (sifakas)	*Propithecus coquereli* (Coquerel's sifaka)	
**Lemuridae** (true lemurs)	*Eulemur albifrons* (White-fronted brown lemur)	
	Eulemur collaris (Red-collared lemur)	
	Eulemjur coronatus (Crowned lemur)	
	Eulemur fulvus (Brown lemur)	
	Eulemur hybrids (Hybrid Eulemur)	
	Eulemur macaco flavifrons (Blue-eyed black lemur)	
	Eulemur macaco macaco (Black lemur)	
	Eulemur mongoz (Mongoose lemur)	
	Eulemur rubriventer (Red-bellied lemur)	
	Eulemur rufus (Red-fronted lemur)	
	Eulemur sanfordi (Sanford's lemur)	
	Hapalemur griseus (Eastern lesser bamboo Lemur)	
	Lemur catta (Ring-tailed lemur)	
	Varecia rubra (Red ruffed lemur)	
	Varecia variegata (Black-and-white ruffed lemur)	
**Lorisidae** (loris and pottos)	*Loris tardigradus* (Red slender loris)	
	Nycticebus bengalensis (Bengal slow loris)	
	Nycticebus coucang (Sunda slow loris)	
	Nycticebus pygmaeus (Pygmy slow loris)	
	Periodictius potto (Potto)	

2.3. On average, how much time does an individual staff member spend training strepsirrhines per day?

a) < 30 minutes
b) 30 minutes-1 hour
c) 1-2 hours

a) > 2 hours

2.4. How many total staff participates in training with strepsirrhine species? Please indicate the number in the space given:

2.5. How long are individual training sessions on average?

a) < 5 minutes
b) 5-10 minutes
c) 10-20 minutes
d) > 20 minutes

2.6. On average, how many training sessions does an individual animal have per week?

a) < 2
b) 2-5
c) > 5

2.7. What are the most challenging strepsirrhine species to train at your organization and why? Please write the species names and select one or more answers for each.

a) Safety concerns
b) Progress slowly
c) Very shy
d) Difficult environmental conditions (e.g. low light, inaccessible enclosure)
e) Hyperreactive
f) Unmotivated
g) Other (please specify)

2.8. When looking to encourage a particular behavior, what type of reinforcement do you use? Please select all that apply:

a) Positive reinforcement
b) Negative reinforcement
c) Vocal encouragement
d) Jackpots
e) Other (please specify)

2.9. When looking to discourage a behavior, what technique do you use? Please select all that apply

a) Negative punishment
b) Positive punishment

c) Least Reinforcement Stimulus
d) Time out
e) Other (please specify)

2.10. Choose the training techniques that are utilized at your facility:

a) Shaping through successive approximations
b) Baiting
c) Scanning or free shaping
d) Modeling, manipulation or sculpting technique
e) Mimicry or imitation technique
f) Active desensitization/counter-conditioning
g) Other (please specify)

2.11. What are the behaviors trained?

a) Basics: bridge, station, target, follow target, point follow
b) Shape recognition
c) Scale training
d) Kennel training
e) Shifting/separation (following target or hand cue)
f) Hang or other posture training for physical examination
g) Voluntary restraint to be transported short distances
h) Syringe training to administer medication or fluids
i) Palpation or manipulation for vet examinations
j) Injection training
k) Positional and duration control for Ultra sound or x-rays
l) Blood sample
m) Infant removal
n) Others (please specify)

3. Evaluation process and impact of training

3.1. What are the risks to conducting training with strepsirrhines at your facility (select all that apply)?

a) None
b) Increased staff injury
c) Increased animal injury
d) Research, education or management interference
e) Decreased animal care due to time spent training
f) Changes in animal behavior

g) Changes in animals’ social structure
h) Monetary expense
i) Human habituation
j) Others (specify)

3.2. In your opinion, what have been the benefits of having a training program for strepsirrhne species at your facility (select all that apply)?

a) Increased animal psychological well-being
b) Increased positive human-animal interactions
c) Increased efficiency in husbandry management
d) Increased efficiency in veterinary care
e) Increased staff awareness of animals’ behaviors
f) Enhanced education of the public
g) Increased staff communication
h) Decreased stress-related behaviors
i) Decreased human-directed aggression
j) Others (please specify)

3.3. How are training sessions documented at your facility (select all that apply)?

a) Paper sheets filled by trainers
b) Electronic sheets filled by trainers
c) Videos
d) Photos
e) There is no record keeping
f) Computer applications for training documentation (ZIMS, TRACKS)
g) Other (please specify)

3.4. How does your program maintain consistency across multiple animals and multiple trainers? (please select all that apply)

a) We recorded data and documented information such as number and duration of sessions, shaping plan steps trained, name of the trainer and animal´s response.
b) We have a criteria to determine if the animal has learned the shaping plan step.
c) We review data from the sessions and create documents to ensure efficiency of the program
d) We have regular meetings to share information
e) We invite speakers or the staff attends professional meetings
f) Others (please specify)

3.5. Do you have a formal process to assess personality or temperament of the strepsirrhines trained or those who are going to be trained?

a) Yes
b) No

If you answered "a" to 3.5, please indicates the way you measured personality or temperament:

a) Behavioral coding (observe animals and record for example frequency and duration of behaviors)
b) Rating personality traits (people familiar with the animals rate them on a set of predefined traits or adjectives on a scale, for example from strongly represented to not represented)
c) Behavioral tests (coding or rating the behavior of the animal in response to a particular situation or experiment, e.g. novelty, aversive stimuli, mirror test)
d) Naturalistic observations (coding or rating the animals over a specific period of time based on their ordinary daily behavior)
e) Cumulative observations (ratings are based on the knowledge and experience that each rater has accumulated because he or she has known the animal)
f) Other (please specify)

3.6. Have you notice differences in strepsirrhine training success according to any of the following? (select all that apply)

a) Sex
b) Housing condition
c) Group structure
d) Social rank
e) Species
f) Personality
g) Other (please specify)

3.7. Do you monitor the impact of training on animal well-being?

a) Yes
b) No

If you have answered "yes" to 3.7, how do you monitor? Please have in mind that your results must be tied to training sessions. You should have evaluated the impact of training on animal welfare or well-being. For example, have you measured some variable before and after training without any change in the environment? If you have doubts about the link

please do not include it. Measures of cortisol in an animal without being linked with training does not count for this question.

a) Physiological measures (e.g. stress response: hormones levels, heart rate variations; immunological measures; body condition)
b) Behavioral measures
c) Video records to monitor primates when staff are not present
d) Other (please specify)
