# Supplementary material for: Strepsirrhine Primate Training Programs in North American Institutions: Status and Implications for Future Welfare Assessment
Source: Animals (Basel). 2021 Aug 21;11(8):2462. doi: 10.3390/ani11082462 (PMC8388707; doi:10.3390/ani11082462)
Supplement: Supplementary file 1 [file animals-11-02462-s001.zip › animals-1300893-supplementary/S2.pdf]

Supplementary File 2 (S2): Differences in training success in five *Eulemur* species.

This study was performed as part of a Ph.D. dissertation in the North American Studies Program at the University of Alcalá (Madrid, Spain) by the first author (GFL). The research was developed at the Duke Lemur Center (Duke University, Durham, NC, USA) with the help of the second author (MD), Erin Ehmke and Sarah Zehr. The study was approved by the DLC Research Committee (project number MO-3-13-1) and the Duke University and Duke University Medical Center Institutional Animal Care and Use Committee (IACUC; protocol number A058-13-03).

Subjects and housing:

14 lemurs of five different species of the same genus *Eulemur* housed at the Duke Lemur Center (DLC), were recruited for the study (Table S1). All of the animals had never previously received training of any kind and were housed in pairs; each pair had access to two connecting enclosures with a total indoor area of approximately 3 m high x 4.6 m wide x 2 m long and outdoor area of 3 m high x 4.6 m wide x 4 m long. Using shift doors, each animal was separated into a single enclosure for the training sessions with visual access to its social partner through wire mesh separating the enclosures. Ten of the animals were housed in the same building, and two pairs (Dido-Mohawk and Francoise-Pascal) were housed in different buildings. Animals were fed once a day and all had access to daily enrichment. The subjects were not involved in any other scientific protocol during the study period.

Table S1: Subject information

DLC ID	Animal	Sex	Species	Age (years)	Notes	Social partner
5982	Francoise	Female	*E. collaris*	28	CB[1]	Pascal
6899	Pascal	Male	*E. collaris*	20	CB[2]	Francoise
6639	Sekar	Male	*E. coronatus*	18	CB[1]	Female *E. coronatus*
7069	Mosi	Male	*E. coronatus*	3	CB[2]	Female *E. coronatus*
6344	Julio	Male	*E. mongoz*	23	CB[1]	Flor
6102	Flor	Female	*E. mongoz*	27	CB[1]	Julio
6305	Geraldine	Female	*E. collaris*	24	CB[1]	Male *E. flavifrons*
6225	Giscard	Male	*E. collaris*	25	CB[1,] N	Taphenes
6257	Taphenes	Female	*E. albifrons*	25	CB[1], N, Hyb	Giscard
6634	Set	Female	*E. coronatus*	18	CB[1]	Male *E. coronatus*
6081	Matthan	Male	*E. albifrons*	27	Hyb	Kish
6184	Kish	Male	*E. albifrons*	26	CB[1,] N, Hyb	Matthan
6038	Dido	Female	*E. rubriventer*	28	WB	Mohawk
6121	Mohawk	Male	*E. rubriventer*	30	WB	Dido

Notes. CB[1]= captive born at DLC; CB[2]= captive born another institution; WB= wild born but in captivity more than 20 years; N= neutered; Hyb= slight hybrid (93.5% *E. albifrons* , 6.5% *E. sanfordi*).

Training methodology:

Subjects were trained using positive reinforcement training techniques to follow a target around a short simple obstacle course to complete an S shape on the ground of each lemur´s home enclosure. We used preferred foods from animals´ diets as reinforcement and a whistle as the bridging stimulus. Animals were reinforced for successive approaches toward the desired behavior and no lemur was forced to participate in training sessions. The sample was divided into two groups of seven individuals each and groups were trained on alternating days from Monday to Friday during the period from May 5th to June 12th, 2013. Each session lasted around 5 minutes and the same trainer (GFL) was used throughout the study in order to minimize the effect of variable trainer experience. In addition, the lemurs had not had any contact with the trainer prior to the study, which minimized the interaction history effect.

Training criteria:

After 10 training sessions, each lemur's success in achieving the goal was scored. To be able to assign a score to each animal, training was divided into five steps:

1. Remained at ease with the trainer inside the enclosure and took food from the trainer's hand.
2. Remained on the floor with the PVC pipes inside the enclosure and associated the sound of the whistle with the reinforcement (bridge).
3. Touched a stationary target (trainer´s hand) in the vicinity of pipes.
4. Followed the trainer´s hand in an S-shape, with intermittent steps allowed.
5. Continuously followed the trainer´s hand in an S-shape.

A step was considered as having been achieved if the animal performed the desired behavior at least 3 times per training session for two consecutive sessions. The criterion for all the individuals was the same. If a lemur was unable to meet the criterion to move on to the next step, it remained in the same step. At the end of training, each lemur received a score from 1 to 5 according to the highest step achieved.

Statistical analyses:

A Kruskal-Wallis test was carried out to test if there was a correlation between species and training success (measured as the training step achieved by each animal). The Conover-Iman method was employed to test for differences between species groups. To test partner effects, a Mann-Whitney test was used to compare the training step achieved by pairs of animals which were enclosure mates to individuals whose enclosure mate was not included in the study. Sex effects were also tested with a Mann-Whitney test to see if there were differences between the training step completed by males and females. Non-parametric tests were used because small sample sizes preclude assumptions of a normal distribution. Alpha values were set at 0.05 and the XLSTAT 2012 software was used.

Results:

Only one lemur finished the training goal (Step 5, continuously following the trainer's hand in an S-shape around the PVC pipes). One lemur accomplished only Step 1, while six subjects (43%)

did not get past Step 2. Training Steps 3 and 4 were each achieved by 21% (n=3) of study subjects. Regarding the animals' sex (Figure S1), 33% of females reached step 4 and 25% of males reached steps 4 and 5. However, the proportion of males and females that obtained lower score in training success was the same (half the males and half the females achieved only steps 1 and 2). The Mann-Whitney test showed no significant differences between training success in males and females (U= 22, P= 0.786).

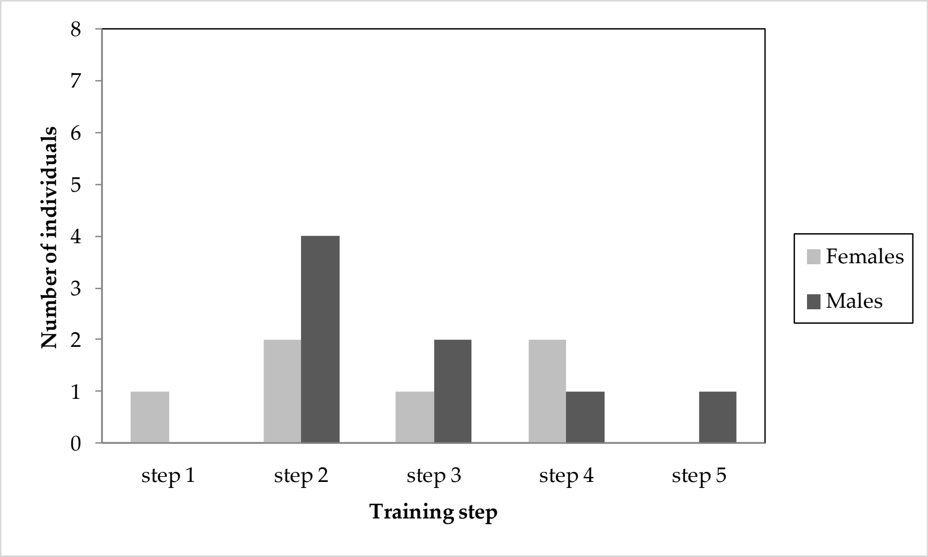

Figure S1: Population distribution according to the sex of the individuals and the step achieved at the end of the 10 training sessions for the 8 males and 6 females in the study.

We found no significant differences between training success in pairs of study animals which were social partners and individuals which had an enclosure mate not included in the study (Mann-Whitney, P= 0.498). We did, however, find significant differences between species and training success (Kruskal-Wallis, k=10.544, df=4, P=0.032). The groups multiple comparison method (Table S2) showed significant differences in training success between E. collaris-E. albifrons (P=0.000), E. collaris-E. rubriventer (P=0.003) and E. mongoz-E. albifrons 275 (P=0.004). The test established three groups (A, B, C, see Figure S2): E. collaris and E. albifrons were the most different in training success with E. collaris (n=4) the species that came the closest to finishing the training goal, while E. albifrons (n=3) was the species that was the farthest from finishing in the 10 training sessions.

Table S2. P-values Conover-Iman multiple comparison method.

	**E. col**	**E. cor**	**E. mongz**	**E. alb**	**E. rub**
E. col	1	0,006	0,505	**0,000**	**0,003**
E. cor	0,006	1	0,042	0,151	0,450
E. mon	0,505	0,042	1	**0,004**	0,018
E. alb	**0,000**	0,151	**0,004**	1	0,554
E. rub	**0,003**	0,450	0,018	0,554	1

Corrected Bonferroni significance level: 0.005

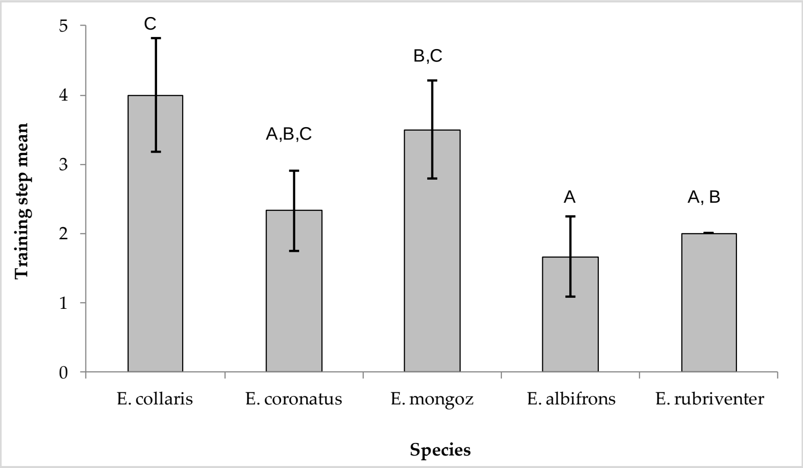

Figure S2: Training success as function of the species of lemur. Each bar represents the mean (± standard error mean; SEM) of the training step achieved by the species at the end of the 10 sessions. Kruskal-Wallis groupings are shown by the letters above each bar. Statistically significant differences ($p < 0.05$) between species are indicated by those that do not share a grouping letter.
